# Creative Thinking in Tourette's Syndrome: An Uncharted Topic

**DOI:** 10.3389/fpsyg.2021.649814

**Published:** 2021-04-22

**Authors:** Laura Colautti, Sara Magenes, Sabrina Rago, Carlotta Zanaboni Dina, Alice Cancer, Alessandro Antonietti

**Affiliations:** ^1^Department of Psychology, Università Cattolica del Sacro Cuore, Milan, Italy; ^2^Fraternità e Amicizia Società Cooperativa Sociale ONLUS, Milan, Italy

**Keywords:** creative thinking, flexibility, tourette's syndrome, assessment, dopamine

## Introduction

This opinion article discusses the literature about creativity in patients with Tourette Syndrome (TS) and provides some suggestions about how this topic might be further exploited. TS is a neurodevelopmental disorder, with an onset occurring before the age of 18 (Robertson, 2011). The main symptom is the presence of tics, of which one or multiple sound tics and multiple motor tics (American Psychiatric Association, 2013). TS is associated with other potential impairments, such as cognitive anomalies, physical pain, impairment in daily activities, emotional and behavioral problems (Cavanna et al., 2013; Morand-Beaulieu et al., 2017).

Despite these problems, high levels of creativity have been reported in TS patients. Examples of eminent individuals, e.g., W. A. Mozart (Ashoori and Jankovic, 2007), allegedly affected by TS, have been described (Szejko et al., 2019). On these premises, researchers studied more recent cases of TS patients showing creative aptitudes and measured the average levels of creative skills in samples of TS patients, to speculate about the reasons why these patients excel in creativity.

## Creativity and Its Neural Correlates

Creativity has been described as a highly functional way of thinking (Dietrich, 2004) leading people to break the usual and automatic responses and to develop alternative behaviors (Heilman, 2016). A “bizarre” idea is not necessarily a creative one; Creative thinking is related to the production of ideas which are both new and appropriate (Runco and Charles, 1993). Creativity includes thinking “beyond borders” enabling people to move away from stereotyped mental schemas (Shamay-Tsoory et al., 2011).

Three neural systems underlie creative thinking (Beaty et al., 2018; Goldberg, 2018): (a) the default network (DN), linked to the generation of ideas through the involvement of mnestic retrieval and mental simulation; (b) the salience network (SN), crucial for the identification of relevant information; (c) the executive control network (ECN), essential for the elaboration, evaluation, and revision of information to maintain a goal-oriented process. Specific neural sites underlie each of these networks: DN involves the ventromedial prefrontal cortex (vmPFC), the orbitofrontal cortex (OFC), the posterior parietal cortex (PPC), and the precuneus. SN includes the insular regions and the anterior cingulate cortex (ACC), whereas ECN encompasses the dorsolateral prefrontal cortex (dlPFC) (Colombo et al., 2015), the ventrolateral prefrontal cortex (vlPFC), and the PPC (Beaty et al., 2018; Goldberg, 2018). The prefrontal cortex (PFC) covers a key role, devoted to the integration of the information, the control of voluntary behaviors, and the inhibition of inadequate responses (Volle et al., 2013; de Souza et al., 2014).

Neurotransmitters are important for creativity as well, in particular dopamine (Lhommée et al., 2014; Khalil et al., 2019). An increase in creative thinking has been reported in Parkinson's Disease (PD) patients after the intake of dopamine drugs (Canesi et al., 2012) and in patients with a pathological excess of dopamine, as occurs in schizophrenia (Folley and Park, 2005; Acar et al., 2018). This is in line with studies showing that dopamine is important for reward drives, curiosity (Flaherty, 2011), and mental associations (McNab et al., 2009), which are linked to creativity.

## TS and Dopamine

The effects of dopamine on creativity are little-known, especially in developmental conditions associated with higher dopamine levels, such as TS. TS is indeed characterized by a dopamine dysfunction due to an atypical neurotransmitter distribution, with an over-activation of dopaminergic circuits in basal ganglia (BG) (Klimkeit and Bradshaw, 2006; Szejko et al., 2019).

The majority of TS symptoms are linked to cortico-striato-thalamo-cortical circuits (which have an important role in communicating with cortical and subcortical structures), and in particular to the frontostriatal system, including dlPFC, lateral OFC, supplementary motor area, ACC, and associated BG structures (Bradshaw and Sheppard, 2000), namely, in brain areas involved in creativity.

In TS, drugs can be prescribed to block the activity of the dopamine receptors, reducing the symptoms during daily activities. Medications can have side effects and, when antidopaminergics are included, a reduction of creativity may occur (Thenganatt and Jankovic, 2016; Porta et al., 2017).

## TS and Creative Thinking

Considering the neural structures involved in TS and the fact that most of them correspond to those involved in creative processes, it has been postulated that the excess of dopamine characterizing TS can enhance creative thinking (Espert et al., 2017). Sacks (quoted in Bradshaw and Sheppard, 2000, p. 307) described TS as “a continuum with two ends: One (stereotypic) extreme involves simple motor tics and vocalizations, iterations, and perseverations, which are largely a nuisance and an irrelevance; The other extreme involves elaborations, playful mimicry, extravagant impudent inventiveness, audacious dramatizations, surreal associations, uninhibited inventiveness, incontinent reactivity”. The second extreme includes a set of manifestations which are associated to creativity.

Single-case studies showed that TS patients have a strong predisposition to, or excel in activities and jobs requiring creativity (Barber, 2016; Porta et al., 2017; Szejko et al., 2019; Zanaboni Dina and Porta, 2019). A recent case is worth mentioning: the soccer player Tim Howard^1^, who developed a personal style of goalkeeping by combining the use of all limbs.

Patients who show higher levels of creativity tend to use TS symptoms to their advantage, obtaining “benefits” from them (Zanaboni Dina and Porta, 2019). Sacks (1992) reported cases of patients who benefited from their symptoms. A TS patient wrote great and brilliant novels, driven by his Tourettic expressive force. A jazz musician was famous for his dynamic improvisations caused by his tics: They were not considered to be mistakes during his performance, but they became its core feature. After a pharmacological treatment, the musician felt calmer and more mindful, but he lost inspiration in improvisation.

Furthermore, regardless of the presence and the type of medication, tics appear to be less intense when TS patients are engaged in creative and artistic performances, such as dancing, painting, or listening to music, but not in other activities (e.g., doing homework, playing videogames) (Caurín et al., 2014).

## Empirical Studies

A search was conducted in Pubmed, PsycINFO, Scopus, and the Cochrane Library databases using the search terms “Tourette's Syndrome” in combination with “creative thinking” and “creativity”. Only two studies investigating creativity in TS using specific tools for the assessment of patients (Wei, 2011; Zanaboni Dina et al., 2017) were found (see Table 1).

**Table 1:** Included studies.

**References**	**Experimental groups**	**Age**	**Creativity assessment**	**Assessed creativity dimensions**	**Results**	**Conclusions/ Hypothesis**
Wei, 2011	Children: 127 TS; 138 CG	TS: m 9.2 ± 1.3 CG: m 9.6 ± 1.5	Williams' Creativity Assessment (Chinese version: Lin and Wang, 1994)	a) Fluency; b) Openness; c) Flexibility; d) Originality, e) Elaboration; f) Naming.	a) TS<CG b) TS<CG c) TS<CG d) TS>CG e) TS<CG^*^ f) TS<CG	No significant differences between the two groups' scores in a), b), c), d), f). This can be explained by the possibility that many TS children have OCD comorbid conditions: Obsessive thoughts and behaviors might limit the elaboration of drawings. Further research is needed.
Zanaboni Dina et al., 2017	Adults: 27 TS; 27 PD	TS: 41.92 ± 6.77 PD: 53.03 ± 5.09	Creative Thinking ASK Test (Italian version: Faraci and Clarotti, 2009)	a) Creative thinking; b) Inventing phrases; c) Producing hypotheses; d) Defining the conditional structure; e) Creating categories.	a) TS>PD^*^ b) TS>PD^*^ c) TS>PD^*^ d) TS>PD e) TS>PD	Results supported the hypothesis that TS group is more prone to be creative than PD group. Important differences have to be considered, such as the age of the two groups, the clinical conditions, the therapy. Further studies are needed.

*TS, Tourette's Syndrome; CG, Control Group; PD, Parkinson's Disease*.

* *p < 0.05*.

In the first study (Wei, 2011) a group of Taiwanese children affected by TS has been matched to a group of typically developing ones. Consistently with the aim of this article, only results related to creative thinking were considered. To assess creativity the Author employed a divergent thinking test composed of 12 drawings that the participants had to complete. TS children got a significant lower score in elaboration, whereas they scored higher in originality, but the difference between the two groups was not statistically significant.

In the second study (Zanaboni Dina et al., 2017), adult TS patients were compared to adults with PD. The two groups were different in age (the TS group was younger than the PD one), in pathology (TS is a neurodevelopmental disorder, PD is a neurodegenerative one), and opposite in therapy (TS therapy usually reduces dopamine levels, PD drugs enhance them). The assessment was based on verbal tasks requiring divergent thinking to process the stimuli. The TS group was more creative in all parameters, even though statistically significant differences emerged only in total creative thinking, inventing phrases, and producing hypotheses scores.

The two studies cannot be compared for two main differences. First, the type of creative task used (figural vs. verbal). Second, the samples' characteristics (study 1: TS children vs. healthy controls; study 2: TS adults vs. PD adults) and medications (study 1: none; study 2: patients undergone different medical therapies or no medications) are dissimilar.

## Discussion

It has been reported that TS patients may exhibit creative attitudes in artistic domains and professional fields in which creativity is required (Sacks, 1992; Porta et al., 2017; Szejko et al., 2019; Zanaboni Dina and Porta, 2019). Studies showed higher scores by TS patients in some dimensions assessed by creativity tests, although differences with matched groups were not always statistically significant (Wei, 2011; Zanaboni Dina et al., 2017). Hence, even if there is no conclusive evidence of an enhanced creativity in TS, data suggest that a creative potential is latent in such a condition.

The creative profile of TS patients can be supported by a possible neurobiological basis. In fact, patterns of enhanced creativity in TS patients can be explained referencing to alterations in dopamine mechanisms. Creative thinking and behavior (i.e., mental associations and curiosity) are fostered by dopaminergic neurotransmission (Lhommée et al., 2014; Boot et al., 2017; Khalil et al., 2019). The hypersensitivity to postsynaptic dopamine receptors (Singer and Walkup, 1991), which characterizes TS, may contribute to the presence of high levels of creativity.

The creative potential in TS patients can be connected to studies using neuroimaging techniques in resting state. TS patients show larger volumes in dorsal PFC and parieto-occipital regions than healthy controls (Peterson et al., 2001), as well as abnormalities in the cortico-striato-thalamo-cortical circuits (Neuner et al., 2010) and altered connectivity patterns between PFC, frontal cortex, midcingulate cortex, precuneus, and inferior parietal gyrus (Church et al., 2009). These areas partially overlap those related to creative thinking, especially PFC, which has a pivotal role in the main mental operations required by creativity.

It has been claimed that creativity plays a crucial role when individuals are challenged, for instance because of neurodevelopmental disorders (Cancer et al., 2016), cognitive impairments (Fusi et al., 2020), brain damages (Colautti et al., 2018), and mental decline associated to aging (Colombo et al., 2018). Original ways to overcome the problems associated to these conditions, when the usual strategies fail, can be found. Hence, creativity can be a resource for TS patients to develop alternative and functional behaviors, helping them cope with their symptoms and promote their well-being.

In addition, the awareness that TS is not associated only to deficits but also to enhanced abilities, such as creativity, can lead patients to develop a more positive representation of themselves. Thus, studying the presence of creativity in TS appears of importance to better understand patients' strengths, integrating into their treatment pathways effective solutions to improve their well-being.

### Future Directions

Further research could investigate creativity (i) across the lifespan; (ii) taking into account social context, parental care, school education, and interactions with peers; (iii) applying standardized approaches. In particular, the use of questionnaires and checklists (i.e., Creative Achievement Questionnaire-CAQ; Carson et al., 2005, or Creative Activity and Accomplishment Checklist-CAAC; Paek et al., 2016) is suggested. Furthermore, studies should consider both the presence of comorbidities, the assumption of drugs, matched controls to make comparisons, and the use of neuroimaging and psychophysiological techniques to investigate the brain circuitry. Genetic bases of TS might be associated to creativity levels as well. Tools which allow a systematic measure of creativity, rather than narrative reports, can provide a more reliable framework. Administering the most used tests to assess creativity (i.e., Torrance Test of Creative Thinking-TTCT; Torrance, 1998) and/or specific mechanisms (i.e., remote associations, Bowden and Jung-Beeman, 2003, or insight, Iannello et al., 2020) is preferable. This should make the comparison of TS findings with those on other pathologies possible. Furthermore, since creativity involves not only divergent thinking but also convergent thinking, further studies should consider both processes. Also, the assessment of personality traits may show if specific personality traits in TS patients are associated with creativity (Abdullah et al., 2016).

Finally, the application of training programs to improve creativity could be addressed to TS patients (i.e., Zanaboni Dina et al., 2020), revealing functional capabilities that would otherwise remain unexploited, and thus compensating possible decreases in creativity induced by the antidopaminergic drugs.

## Author Contributions

LC and SM conceived the ideas presented. LC, SM, and SR wrote the article. SR formatted the article. AC revised the language. CZ, AA, and AC critically revised the manuscript. All authors contributed to the article and approved the submitted version.

## Conflict of Interest

The authors declare that the research was conducted in the absence of any commercial or financial relationships that could be construed as a potential conflict of interest.

## Footnotes

^1^https://www.youtube.com/watch?v=9IaSOktjtv4
